# Interleukin-4-Mediated Oxidative Stress Is Harmful to Hippocampal Neurons of Prothrombin Kringle-2-Lesioned Rat In Vivo

**DOI:** 10.3390/antiox9111068

**Published:** 2020-10-30

**Authors:** Young Cheul Chung, Jae Yeong Jeong, Byung Kwan Jin

**Affiliations:** 1 Department of Predictive Toxicology, Korea Institute of Toxicology, Daejeon 34114, Korea; youngcheul.chung@kitox.re.kr; 2 Department of Biochemistry & Molecular Biology, School of Medicine, Kyung Hee University, Seoul 02447, Korea; jyoung0229@khu.ac.kr; 3 Department of Neuroscience, Graduate School, Kyung Hee University, Seoul 02447, Korea

**Keywords:** oxidative/nitrosative stress, neurodegeneration, microglia/macrophages, prothrombin-kringle-2, interkeukin-4

## Abstract

The present study investigated the effects of reactive microglia/macrophages-derived interleukin-4 (IL-4) on hippocampal neurons in prothrombin kringle-2 (pKr-2)-lesioned rats. pKr-2 was unilaterally injected into hippocampus in the absence or presence of IL-4 neutralizing antibody (IL-4Nab). Immunohistochemical analysis showed a significant loss of Nissl^+^ and NeuN^+^ cells and activation of microglia/macrophages (increase in reactive OX-42^+^ and OX-6^+^ cells) in the hippocampus at 7 days after pKr-2 injection. The levels of IL-4 expression were upregulated in the reactive OX-42^+^ microglia/macrophages as early as 1 day, maximal at 3 days and maintained up to 7 days after pKr-2 injection. Treatment with IL-4Nab significantly increased neuronal survival in pKr-2-treated CA1 layer of hippocampus in vivo. Accompanying neuroprotection, IL-4 neutralization inhibited activation of microglia/macrophages, reactive oxygen species-derived oxidative damages, production of myeloperoxidase- and inducible nitric oxide synthase-derived reactive nitrogen species and nitrosative damages as analyzed by immunohistochemistry and hydroethidine histochemistry. These results suggest that endogenous IL-4 expressed on reactive microglia/macrophages mediates oxidative/nitrosative stress and play a critical role on neurodegeneration of hippocampal CA1 layer in vivo.

## 1. Introduction

Oxidative/nitrosative stress, defined as ‘an imbalance in pro-oxidants and antioxidants with associated disruption of redox circuitry and macromolecular damage’ is strongly associated with the development of neuronal death and dysfunction [1,2]. The balance between reactive oxygen species/reactive nitrogen species (ROS/RNS) production and the antioxidant defense is modulated by multiple sources such as ROS/RNS production enzymes and free radical scavengers to maintain cellular functions [1,3]. Accumulating evidence suggests that oxidative/nitrosative stress is well-known feature of neurodegenerative disorders, including Alzheimer’s disease (AD), Parkinson’s disease (PD) and amyotrophic lateral sclerosis (ALS) [1,4,5]. Accordingly, antioxidants may be beneficial for the treatment of neurodegenerative disorders, that are associated with oxidative damage.

Microglia, resident macrophages in the brain, are known to neuronal support cells that maintain synaptic remodeling and brain homeostasis in healthy conditions [6,7]. Under pathological conditions, microglia are transformed to become reactive and then to determine whether a neuron should be cleared (neurotoxic) or salvageable (neurotrophic) [7,8]. In a neurodegenerative condition, reactive microglia preserve a neurotoxic state through triggering production of inflammatory molecules and oxidative/nitrosative stress [4,6,7,8].

Prothrombin kringle-2 (pKr-2), the second domains of prothrombin which is generated by active thrombin, is known to directly induce microglial activation and produce neurotoxic molecules such as inducible nitric oxide synthase (iNOS)-derived ROS/RNS, tumor necrosis factor-alpha (TNF-α) and interleukin-1beta (IL-1β) in vivo and in vitro [9,10,11]. pKr-2-induced microglial activation and neurotoxic molecules contribute to neurodegeneration in the cortex and nigrostriatal dopamine system in vivo [9,10,12]. In addition, up-regulated levels of pKr-2 and thrombin were observed in the postmortem brains of AD and PD patients, indicating the relevance of pKr-2 in the neurodegenerative disorders [12,13].

Interleukin-4 (IL-4) is well-known anti-inflammatory cytokine and has diverse immunomodulatory functions on cell repair and regeneration in the central nervous system [14,15]. In the context of neuroinflammation under pathological conditions, it exerts either harmful or beneficial effects on neurons by regulating oxidative stress in vivo and in vitro [9,11,15,16,17]. Thus, the present study examined whether pKr-2-induced neurotoxicity could contribute to endogenous production of IL-4, which is neurotoxic or neuroprotective in hippocampus in vivo.

## 2. Materials and Methods

### 2.1. Chemicals

Prothrombin kringle-2 (pKr-2) was purchased from Haematologic Technologies (Essex Junction, VT, USA). Interleukin-4 neutralizing antibody (IL-4Nab) and non-specific IgG were obtained from R&D system (Minneapolis, MN, USA), and dihydroethidium (hydroethidine) was purchased from ThermoFisher Scientific (Waltham, MA, USA).

### 2.2. Animals

Animal experiments were carried out according to the guideline set by Committee on Animal Research of Kyung Hee University (KHUASP-20-235, 30 June 2020). Female Sprague-Dawley rats were used in this study. They were housed three per cage under a 12 h light/dark cycle in a temperature-controlled room (21–23 °C). Throughout the experiment, food and water were available ad libitum. All efforts were made to minimize animal suffering and the minimal number of animals necessary to produce reliable scientific data were used.

### 2.3. Intrahippocampal Microinjection

As described in References [9,18], rats weighing 240–260 g (10 weeks of age) were anesthetized with intraperitoneally (i.p.) administered chloral hydrate (360 mg/kg; Sigma-Aldrich., St. Louis, MO, USA) and placed in a stereotaxic apparatus. The pKr-2 (48 μg in 4 μL phosphate-buffered saline (PBS; Gibco., Paisley, UK)) was unilaterally injected into right CA1 layer of hippocampus (3.6 mm posterior to bregma, 2.0 mm lateral to the midline, 2.8 mm beneath the skull of the brain according to the atlas of Paxinos and Watson [19] with 30 gauge of Hamilton syringe at a rate of 0.2 μL per minute. For neutralization of interleukin-4 (IL-4) [9,18], the mixture of pKr-2 and IL-4Nab (1 μg/μL) or non-specific IgG as a control (1 μg/μL) was injected into the equivalent coordinate of right CA1 layer of hippocampus.

### 2.4. Immunohistochemistry (IHC) and Immunofluorescence (IF) Staining

Animals were deeply anesthetized with an overdose of chloral hydrate (intraperitoneally) and perfused transcardially with physiological saline containing 0.5% sodium nitrate and heparin (10 U/mL), followed by 4% paraformaldehyde in 0.1 M phosphate buffer (pH 7.4). Brains were then removed, post-fixed overnight and transferred to 30% sucrose solution. Serial coronal sections were cut sectioned at 40 μm thickness on a sliding microtome. Every sixth section was processed for immunohistochemical staining as described [9,20]. In brief, free-floating brain sections were rinsed with PBS, quenched in PBS containing 3% H_2_O_2_, blocked in PBS containing 1% bovine serum albumin (BSA; Millipore corporation., Kankakee, IL, USA) with 0.2% triton X-100 (Sigma-Aldrich), and then incubated overnight at room temperature with primary antibodies (Table 1). The following day, brain sections were rinsed in PBS containing 0.5% BSA, incubated with the biotin-conjugated mouse IgG (Table 2), processed with an avidin-biotin complex kit (Vector Laboratories., Burlingame, CA, USA) and visualized 0.05% 3,3′-diaminobenzidine tetrahydrochloride hydrate (DAB; Sigma-Aldrich) and 0.003% hydrogen peroxide. Labeled tissue sections were then mounted on adhesive microscope slides and viewed under a bright-field microscope (Olympus BX51; Olympus Optical., Tokyo, Japan). The primary antibodies used were as follows: mouse anti-OX-42 for microglia/macrophages and neutrophils, mouse anti-OX-6 for activated microglia, mouse anti-neuron-specific nuclear protein (NeuN) for neurons and mouse anti-8-hydroxy-2-deoxy guanosine (8-OHdG) for detecting oxidative DNA damage (Table 1).

For Nissl staining, some of the hippocampal tissues were mounted on adhesive microscope slides, dried, stained in 0.5% cresyl violet acetate solution (Sigma-Aldrich), dehydrated, covered, and then analyzed under a bright-field microscope.

For immunofluorescence staining, tissue sections were processed as described [9,20]. Free-floating sections were mounted on adhesive microscope slides and dried at room temperature. After washing, sections were incubated with primary antibodies overnight at 4 °C (Table 1). The following day, brain sections were rinsed in PBS containing 0.5% BSA, incubated with the appropriate fluorescence-conjugated secondary antibodies. The sections were washed, covered with vectashield mounting medium for fluorescence with DAPI (Vector Laboratories), and analyzed under confocal microscope (LSM700; Carl Zeiss., Oberkochen, Germany). The primary antibodies used were as follows: rabbit anti-IL-4, fluorescein isothiocyanate (FITC)-labeled tomato lectin (TL) and mouse anti-OX-42 for microglia/macrophages and neutrophils, NeuN, mouse anti-glial fibrillary acidic protein (GFAP) for astrocytes, rabbit anti-myeloperoxidase (MPO), rabbit anti-inducible nitric oxide (iNOS), and mouse anti-nitrotyrosine for recognizing ROS/RNS-dependent protein damage (Table 1). The fluorescence-conjugated secondary antibodies used were as follows: FITC-conjugated anti-mouse, fluorescein-conjugated anti mouse, and Cy3-conjugated anti-rabbit (Table 2).

### 2.5. In Situ Detection of O_2_^−^ and O_2_^−^-Derived Oxidants

In situ O_2_^−^ production was visualized by hydroethidine histochemistry [9,20]. At 3 days after pKr-2 injection, rats received intravenous injection of hydroethidine (1 mg/kg in PBS containing 1% dimethyl sulfoxide) into tail vein. After 45 min, rats were transcardially perfused and fixed. Brain tissues were cut sectioned at 40 μm thickness on a sliding microtome and mounted on adhesive microscope slides. The oxidized hydroethidine product, ethidium, was investigated by confocal microscopy (LSM700) and merged with DAPI solution (Vector Laboratories).

### 2.6. Quantification of Neurons in Hippocampal CA1 Layer

To quantify the number of hippocampal neurons in CA1 layer of hippocampus, neurons were counted on four levels of the dorsal hippocampus, respectively. Specifically, alternate sections were obtained from the following coordinates: 3.3, 3.6, 4.16, and 4.3 mm posterior to bregma (totally, 4 areas for each animal) were used to count cells in the CA1 layer. Using a bright-field microscope at a magnification of 40X, the number of neurons in the CA1 layer was counted and quantified as the number of CA1 per millimeter of linear length (Hippocampal CA1 neurons/mm), as described previously [18,21]. To maintain consistency between each animal, we counted the number of neurons from 1.5 mm lateral to the midline. Only neurons with normal visible nuclei were counted. The mean number of CA1 neurons per millimeter in 4 areas for each animal was calculated for each experimental group.

### 2.7. Image J Analysis

To analyze and quantify the expression of IL-4, 8-OHdG, MPO, iNOS, nitrotyrosine and oxidized hydroethidine product in pKr-2-injected CA1 layer of hippocampus with absence or presence of IL-4Nab, images were obtained from the same area in each tissue sample. Imaging data were analyzed as pixel value, the number of pixels above the threshold, through Image J (National Institutes of Health). Image J was used to quantify chromogenic signal intensity of immunofluorescence on image. All original images were collected, converted to 8-bit grayscale and the minimum threshold values of images were adjusted at the endpoint of histogram for excluding the background signal. Then, the signals were quantified as pixel values and normalized by unstained area.

### 2.8. Statistical Analysis

Results are expressed as means ± SEM. For statistical evaluation, the data was assessed by one-way ANOVA Newman–Keuls analyses and Student’s unpaired *t*-test (GraphPad Software, San Diego, CA, USA). Significance level was set at *p* < 0.05.

## 3. Results

### 3.1. pKr-2 Induces Activation of Microglia/Macrophages and Neuronal Death in the Hippocampus In Vivo

**Figure 1:**
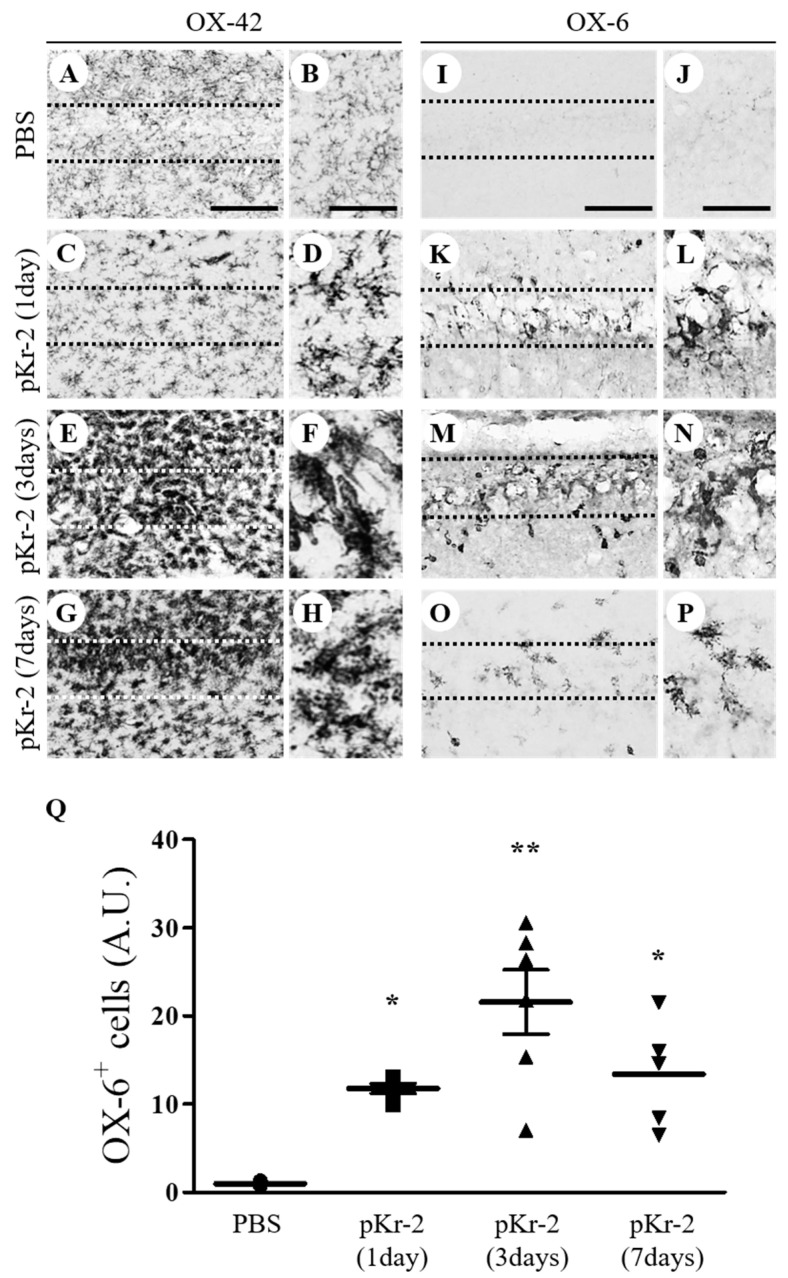
Prothrombin kringle-2-induced microglial/macrophages activation in the CA1 area of the hippocampus in vivo. Phosphate-buffered saline (PBS) (**A**,**B**,**I**,**J**) as a control or prothrombin kringle-2 (pKr-2; **C**–**H**, **K**–**P**; 48 μg/ 4 μL) was unilaterally injected into the CA1 layer of rat hippocampus. Animals were transcardially perfused at indicated time points and brain tissues were prepared for immunohistochemical staining. Every sixth serial sections were selected and immunostained with OX-42 (complement receptor 3, CR3; **A**–**H**) to identify microglia/macrophages and neutrophils or OX-6 (major histocompatibility complex class II; **I**–**P**) to identify activated microglia at 1 day (**C**,**D**,**K**,**L**), 3 days (**E**,**F**,**M**,**N**) and 7 days (**G**,**H**,**O**,**P**) after intrahippocampal injection of pKr-2. (**B**,**D**,**F**,**H**,**J**,**L**,**N** and **P**) show higher magnifications of (**A**,**C**,**E**,**G**,**I**,**K**,**M** and **O**), respectively. Scale bars, 100 μm (**A**,**C**,**E**,**G**,**I**,**K**,**M** and **O**), 100 μm (**B**,**D**,**F** and **H**) and 50 μm (**J**,**L**,**N** and **P**). (**Q**) Quantification of OX-6^+^ cells in the CA1 layer of pKr-2-injected hippocampus. * *p* < 0.01, ** *p* < 0.001 significantly different from PBS. mean ± SEM; *n* = 5 to 7 in each group, ANOVA and Newman–Keuls analysis. Note the morphological changes from resting (small cell bodies with ramified processes) to activated state (large cell bodies with short processes) in the pKr-2-injected CA1 layer of hippocampus, compared with the PBS-injected control.

Recent findings, including ours, indicate that pKr-2 activates microglia in the cerebral cortex and substantia nigra in vivo [9,11]. The present study determined the activation of microglia/macrophages by pKr-2 in the CA1 layer of hippocampal neurons in vivo. To test this, PBS as a control or pKr-2 (48 μg/4 μL) was unilaterally injected into the CA1 layer of rat hippocampus. Immunohistochemical analysis demonstrated that OX-42-immunopositive (OX-42^+^) microglia/macrophages exhibited a resting state, small cell bodies with ramified processes in the PBS-injected CA1 layer at 7 days after injection (Figure 1A,B). In contrast, the majority of OX-42^+^ microglia/macrophages displayed an activated state, large cell bodies with short, thick, or no processes, which were observed as early as 1 day after pKr-2 injection (Figure 1C,D) and sustained up to 7 days after pKr-2 injection (Figure 1E–H). Similar to changes of OX-42^+^ cells, many OX-6^+^ cells were observed in the CA1 layer of hippocampus as early as 1 day after pKr-2 injection (Figure 1K,L,Q) and maintained up to 7 days after pKr-2 injection (Figure 1M–Q), whereas in PBS-injected control, few of OX-6^+^ cells were seen (Figure 1I,J,Q).

**Figure 2:**
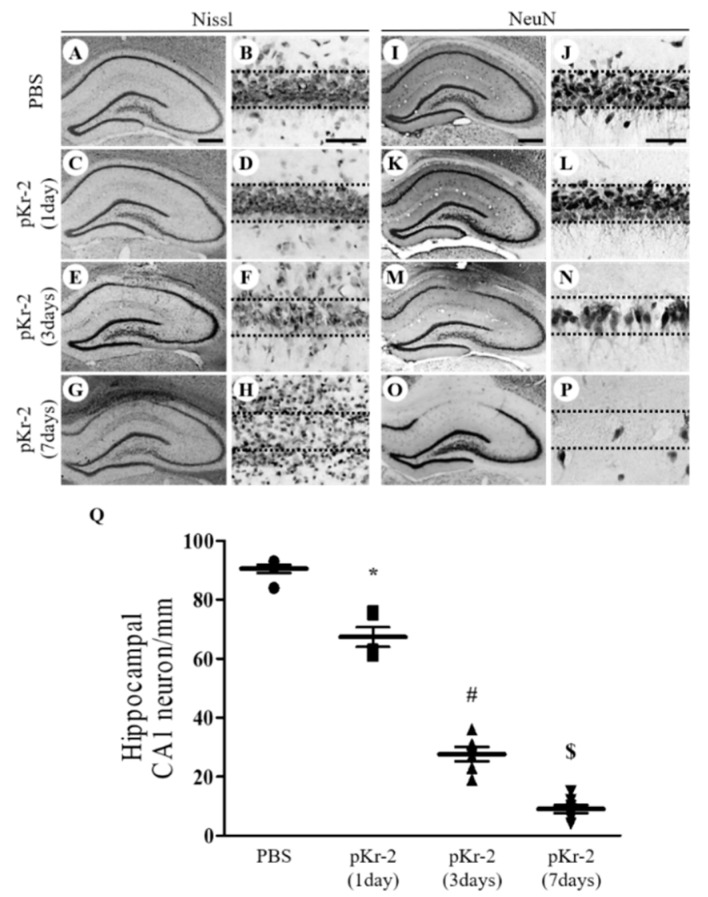
pKr-2-induced neurotoxicity in the CA1 layer of the hippocampus in vivo. Sections (**A**,**B**,**I**,**J**, PBS; **C**–**H, K**–**P**, pKr-2) adjacent to those used in Figure 1 were processed for Nissl staining or neuronal nuclei (NeuN) immunostaining at indicated time points. (**B**,**D**,**F**,**H**,**J**,**L**,**N** and **P**) show higher magnifications of (**A**,**C**,**E**,**G**,**I**,**K**,**M** and **O**), respectively. (**A**–**H**) CA1 layer of the hippocampus stained for Nissl substance (cresyl violet). Scale bars, 500 μm (**A**,**C**,**E** and **G**), 50 μm (**B**,**D**,**F** and **H**). (**I**–**P**) NeuN immunostaining in the CA1 layer of hippocampus. Scale bars, 500 μm (**I**,**K**,**M** and **O**), 50 μm (**J**,**L**,**N** and **P**). (**Q**) Quantification of NeuN^+^ cells in the CA1 layer of pKr-2-injected hippocampus. * *p* < 0.001, significantly different from PBS. ^#^ *p* < 0.001, significantly different from pKr-2 (1day). ^$^ *p* < 0.001, significantly different from pKr-2 (3days). mean ± SEM; *n* = 5 to 7 in each group, ANOVA and Newman–Keuls analysis.

As pKr-2 induces neurodegeneration in the cerebral cortex and substantia nigra [9,10,11], we hypothesized that pKr-2 could produce neurodegeneration in the CA1 layer of the hippocampus. Sections adjacent to those used for Figure 1 were processed for Nissl staining and immunostaining for neuronal nuclei (NeuN) to detect general hippocampal neurons. Compared to PBS-injected control (Figure 2A,B), a significant reduction of Nissl-stained cells was induced as early as 1 day after pKr-2 injection (Figure 2C,D), increased at 3 days after pKr-2 injection (Figure 2E,F) and maximal showing marked loss of Nissl substances with severe gliosis at 7 days after pKr-2 injection (Figure 2G,H). NeuN immunohistochemical staining showed that a significant loss of NeuN^+^ cells was seen in the CA1 layer of hippocampus as early as 1 day after pKr-2 injection (Figure 2K,L,Q), increased at 3 days after pKr-2 injection (Figure 2M–Q) and maximal at 7 days after pKr-2 injection (Figure 2O,P,Q) compared to PBS-injected control (Figure 2I,J,Q). Taken together, these results indicate that pKr-2 induces activation microglia/macrophages as well as neurodegeneration in the CA1 layer of hippocampus in vivo in a time-dependent manner.

### 3.2. Endogenous IL-4 Expressed within Reactive Microglia/Macrophages Contributes to Neurodegeneration in pKr-2-Injected CA1 Layer of Hippocampus In Vivo

**Figure 3:**
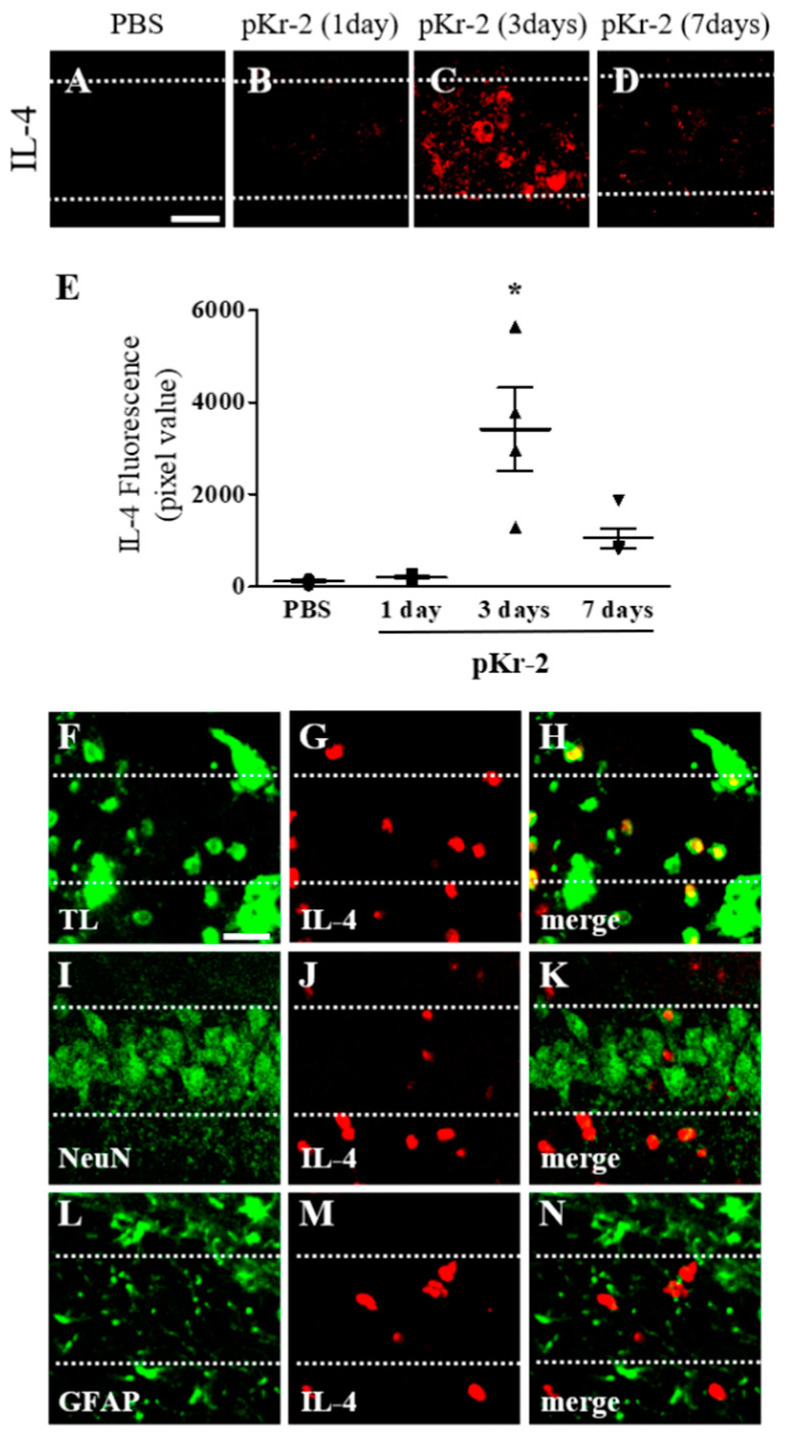
Expression of endogenous interleukin-4 in the CA1 layer of pKr-2-lesioned hippocampus in vivo. PBS (**A**) or pKr-2 (**B**–**D**, **F**–**N**) was unilaterally injected into the CA1 layer of rat hippocampus. Animals were transcardially perfused at various time points and brain sections were processed for immunohistochemical staining. (**A**–**E**) Immunofluorescence images of IL-4 (**A**–**D**) and quantification (**E**) in the CA1 layer of rat hippocampus at the indicated time points. * *p* < 0.001, significantly different from PBS (control). mean ± SEM; E, *n =* 4 to 5 in each group, ANOVA and Newman–Keuls analysis. Scale bar, 40 μm. (**F**–**N**) Double immunofluorescence images of tomato lectin (TL; **F**, green) for microglia/macrophages and IL-4 (**G**, red) or neuronal nuclei (NeuN; **I**, green) for neurons and IL-4 (**J**, red) or glial fibrillary acidic protein (GFAP; **L**, green) for astrocytes and IL-4 (**M**, red) and both images are merged (yellow; **H**,**K** and **N**) in the CA1 layer of hippocampus at 3 days after pKr-2 injection. Scale bar, 25 μm. These data are representative of four to five animals per group. Dotted lines indicate the CA1 layer of the hippocampus.

IL-4 expressed in CD11b^+^-reactive microglia/macrophages contributes to degeneration of neurons in the pKr-2-treated cortex in vivo [9] and in the thrombin- or beta-amyloid_1-42_-treated hippocampus in vivo [18,22]. Accordingly, we next examined whether pKr-2 could induce expression of IL-4 within reactive microglia/macrophages in the CA1 layer of hippocampus, resulting in neurodegeneration. Immunohistochemical analysis demonstrated that endogenous expression of IL-4 was observed as early as 1 day after pKr-2 injection (Figure 3B,E), maximal at 3 days after pKr-2 injection (Figure 3C,E) and sustained up to 7 days after pKr-2 injection (Figure 3D,E) compared to PBS-injected control (Figure 3A,E).

Expression of IL-4 was analyzed in tomato lectin (TL)^+^ microglia/macrophages, NeuN^+^ neurons and GFAP^+^ astrocytes at 3 days after pKr-2 injection. Expression of IL-4 was mainly localized in TL^+^ microglia/macrophages (Figure 3F–H), but neither in NeuN^+^ neurons (Figure 3I–K) nor GFAP^+^ astrocytes (Figure 3L–N) in CA1 layer of hippocampus in vivo.

**Figure 4:**
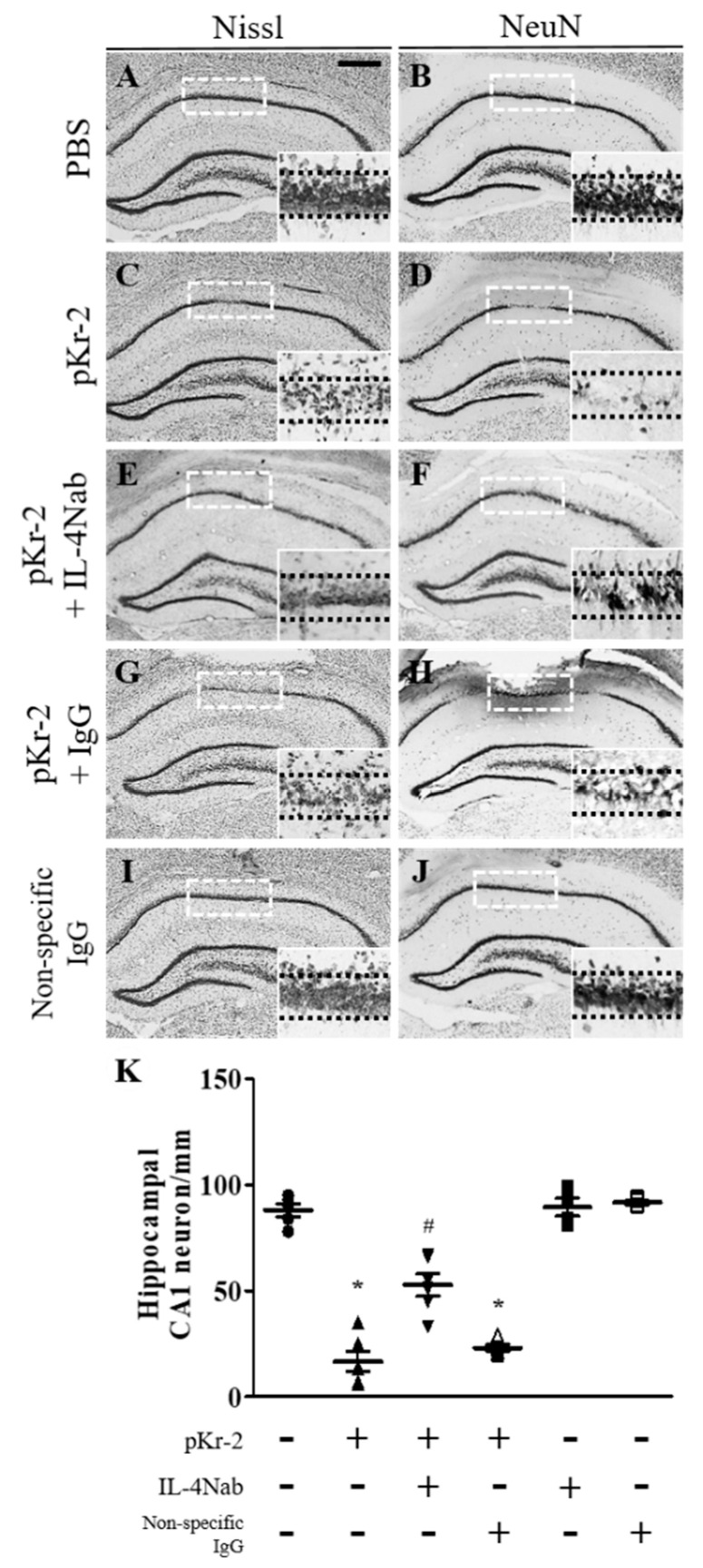
IL-4-mediated neurotoxicity in the CA1 layer of pKr-2-injected hippocampus in vivo. Animals unilaterally received intrahippocampal injection of PBS (**A**,**B**) as a control, pKr-2 (**C**,**D**), pKr-2 + IL-4Nab (1 μg; **E**,**F**), pKr-2 + non-specific IgG (1 μg; **G**,**H**), and non-specific IgG only (1 μg; **I**,**J**). At 3 days after injection, animals were transcardially perfused and brain tissues were processed for Nissl staining or neuronal nuclei (NeuN) immunostaining at 3 days after pKr-2 injection. (**A**,**C**,**E**,**G**,**I**) CA1 layer of the hippocampus stained for Nissl substance. (**B**,**D**,**F**,**H**,**J**) NeuN immunostaining in the CA1 layer of hippocampus. Insets show magnified photomicrographs in the CA1 layer marked by dotted rectangles, respectively. Scale bar, 500 μm. (**K**) Number of NeuN^+^ cells in the CA1 layer of pKr-2-injected hippocampus in the absence or presence of IL-4Nab. * *p* < 0.001, significantly different from PBS. ^#^ *p* < 0.001, significantly different from pKr-2. mean ± SEM; *n* = 4 to 6 in each group, ANOVA and Newman–Keuls analysis.

**Figure 5:**
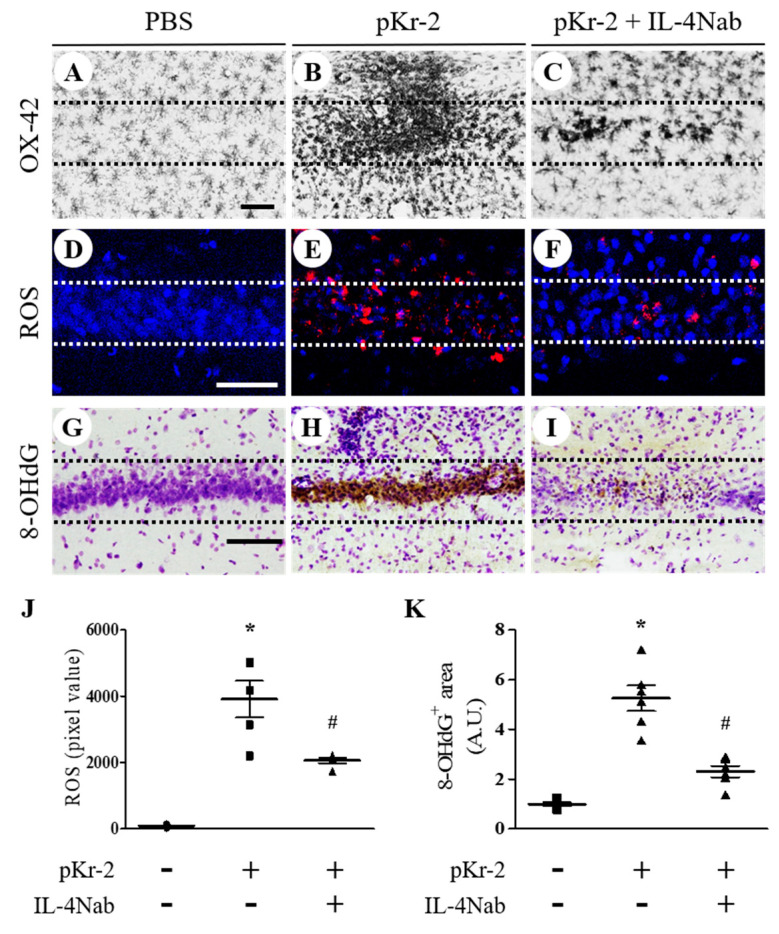
IL-4 induces activation of microglia/macrophages and oxidative stress in the CA1 layer of pKr-2-injected hippocampus in vivo. Sections (**A**,**D**,**G** (PBS); **B**,**E**,**H** (pKr-2); **C**,**F**,**I** (pKr-2 + IL-4Nab)) adjacent to those used in Figure 4 were processed for immunohistochemical staining or hydroethidine histochemistry. (**A**–**C**) CA1 layer of the hippocampus stained for OX-42 antibody to identify microglia/macrophages. Scale bar, 100 μm. (**D**–**F**) Hydroethidine histochemistry to detect oxidant production (ethidium fluorescence, red) in the CA1 layers of hippocampus. Nuclei were counterstained with DAPI (blue). Scale bar, 50 μm. (**G**–**I**) CA1 layers of the hippocampus stained for 8-hydroxy-2-deoxy guanosine (8-OHdG) antibody to detect oxidative DNA damages and counterstained with Nissl. Scale bar, 100 μm. (**J**) Quantification of ROS expression. ^*^ *p* < 0.001, significantly different from PBS (control). ^#^ *p* < 0.001, significantly different from pKr-2. mean ± SEM; *n =* 4 to 5 in each group, ANOVA and Newman–Keuls analysis. (**K**) Quantification of 8-OHdG expression. ^*^ *p* < 0.001, significantly different from PBS. ^#^ *p* < 0.001, significantly different from pKr-2. mean ± SEM; *n =* 4 to 5 in each group, ANOVA and Newman–Keuls analysis. Dotted lines indicate the CA1 layer of the hippocampus.

Next, IL-4-mediated neurotoxicity was assessed in CA1 layer of hippocampus in vivo at 3 days after pKr-2 injection. Immunohistochemical analysis exhibited the significant loss of Nissl^+^ and NeuN^+^ (Figure 4C,D,K) cells in pKr-2-injected CA1 layer of hippocampus compared to PBS-injected control (Figure 4A,B,K), respectively. To test IL-4-mediated neurodegeneration, IL-4 neutralizing antibody (IL-4Nab) was unilaterally co-injected with pKr-2 to block the action of IL-4 into the CA1 layer of rat hippocampus. Immunohistochemical analysis showed that treatment of IL-4Nab significantly prevented pKr-2-induced degeneration of Nissl^+^ and NeuN^+^ (Figure 4E,F,K) cells in the CA1 layer of the hippocampus. Treatment with IL-4Nab alone did not affect neuronal survival (Figure 4K). As a control, non-specific goat IgG with (Figure 5G,H,K) or without pKr-2 (Figure 5I–K) had little effect, similar to that observed with injection of pKr-2 only.

### 3.3. IL-4 Induces Activation of Microglial/Macrophages, ROS Production and Oxidative Damages in pKr-2-Injected CA1 Layer of Hippocampus In Vivo

We previously reported that IL-4 regulates activation of microglia/macrophages in the pKr-2-treated cortex and lipopolysaccharide (LPS)-treated substantia nigra in vivo [9,23]. Accordingly, we examined whether IL-4 could activate microglia/macrophages in the CA1 layer of hippocampus in vivo at 3 days after pKr-2 injection. Immunohistochemical analysis demonstrated that the majority of OX-42^+^ microglia/macrophages were activated in pKr-2-injected CA1 layer of hippocampus (Figure 5B) compared to PBS-injected hippocampus (Figure 5A). Treatment of IL-4Nab prevented pKr-2-induced activation of OX-42^+^ microglia/macrophages in the CA1 layer of hippocampus (Figure 5C).

As IL-4 mediates O_2_^−^ production in the thrombin- and beta-amyloid_1-42_–lesioned hippocampus or pKr-2-lesioned cortex [9,18,22], we hypothesized that IL-4 could contribute to O_2_^−^ production in pKr-2-treated hippocampus in vivo. To test this, hydroethidine histochemistry was performed for in situ visualization of O_2_^−^ production. The fluorescent products of oxidized hydroethidine (ethidium accumulation; red fluorescence) were increased 3 days after pKr-2 injection (Figure 5E,J) compared to PBS-injected control (Figure 5D,J). Treatment of IL-4Nab significantly reduced pKr-2-induced O_2_^−^ production in the CA1 layer of the hippocampus (Figure 5F,J).

To address the role of IL-4 on oxidative DNA damage, 8-OHdG levels were measured in pKr-2-injected CA1 layer of hippocampus in the presence or absence of IL-4Nab. Immunohistochemical analysis showed that pKr-2 injection increased levels of 8-OHdG in the CA1 layer of hippocampus at 3 days after pKr-2 injection (Figure 5H,K), compared to PBS-injected control (Figure 5G,K). Treatment of IL-4Nab significantly reduced pkr-2-induced 8-OHdG levels in the CA1 layer of hippocampus (Figure 5I,K).

### 3.4. IL-4 Induces Oxidative/Nitrosative Stress in pKr-2-Injected CA1 Layer of Hippocampus In Vivo through MPO and iNOS

**Figure 6:**
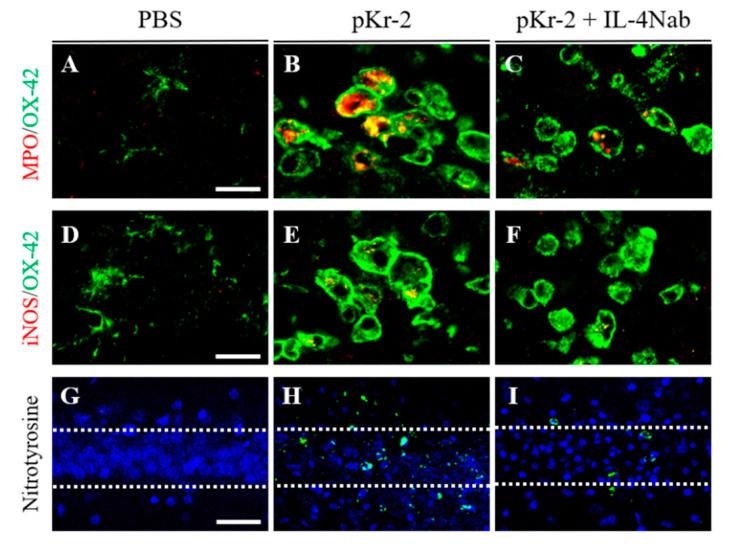

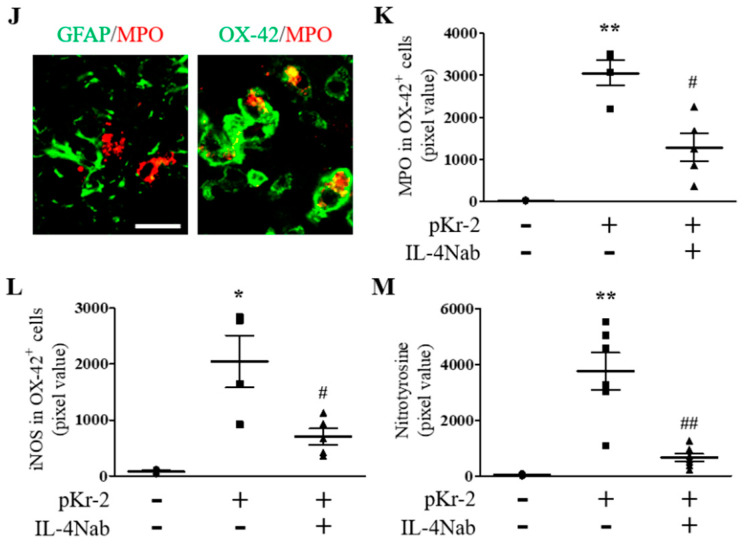
IL-4 modulates MPO- and iNOS-derived oxidative/nitrosative stress within activated microglia/macrophages in the CA1 layer of pKr-2-injected hippocampus in vivo. Sections (**A**,**D**,**G** (PBS); **B**,**E**,**H** (pKr-2); **C**,**F**,**I** (pKr-2 + IL-4Nab)) adjacent to those used in Figure 4 were processed for immunohistochemical staining. (**A**–**C**) Double immunofluorescence images of myeloperoxidase (MPO; red) and OX-42 (green) for microglia/macrophages/neutrophils and both images are merged (yellow) in the CA1 layer of hippocampus. Scale bar, 20 μm. (**D**–**F**) Double immunofluorescence images of inducible nitric oxide synthase (iNOS; red) and OX-42 (green) for microglia/macrophages/neutrophils and both images are merged (yellow) in the CA1 layer of hippocampus. Scale bar, 20 μm. (**G**–**I**) Immunofluorescence images of nitrotyrosine (green) to detect nitrosative damages in the CA1 layer of hippocampus. Nuclei were counterstained with DAPI (blue). Scale bar, 40 μm. (**J**) Double immunofluorescence images of GFAP (green, left panel) for astrocytes and MPO (red, left panel) or OX-42 (green, right panel) for microglia/macrophages/neutrophils and MPO (red, both panel) and both images are merged (yellow) in the CA1 layer of hippocampus. Scale bar, 20 μm. (**K**) Quantification of MPO expression in OX-42^+^ microglia/macrophages/neutrophils. ** *p* < 0.001, significantly different from PBS (control). ^#^ *p* < 0.01, significantly different from pKr-2. mean ± SEM; *n =* 4 to 5 in each group, ANOVA and Newman–Keuls analysis. (**L**) Quantification of iNOS expression in OX-42^+^ microglia/macrophages/neutrophils. * *p* < 0.01, significantly different from PBS (control). ^#^ *p* < 0.01, significantly different from pKr-2. mean ± SEM; *n =* 4 to 5 in each group, ANOVA and Newman–Keuls analysis. (**M**) Quantification of nitrotyrosine expression. ** *p* < 0.001, significantly different from PBS (control). ^##^ *p* < 0.001, significantly different from pKr-2. mean ± SEM; *n* = 4 to 5 in each group, ANOVA and Newman–Keuls analysis. Dotted lines indicate the CA1 layer of the hippocampus.

Many studies demonstrated that myeloperoxidase (MPO) and inducible nitric oxide synthase (iNOS) participated in ROS/RNS production, resulting in neurodegeneration in vivo [1,4]. Moreover, microglia/macrophages and/or neutrophils were found to produce MPO- and iNOS-derived ROS/RNS in vivo [9,24,25,26,27]. Accordingly, we next investigated whether IL-4 could modulate expression of MPO and iNOS within reactive microglia/macrophages/neutrophils in the CA1 layer of hippocampus. Similar to our previous reports [9,24], immunohistochemical analysis showed that expression of MPO and iNOS was significantly increased in OX-42^+^ microglia/macrophages/neutrophils at 3 days after pKr-2-injected CA1 layer of hippocampus in vivo (Figure 6B,E,K,L) compared to PBS-injected control (Figure 6A,D,K,L). In contrast, treatment of IL-4Nab significantly reduced pKr-2-induced MPO and iNOS levels in OX-42^+^ microglia/macrophages/neutrophils in the CA1 layer of hippocampus (Figure 6C,F,K,L). MPO expression is mainly localized in OX-42^+^ microglia/macrophages/neutrophils, but not in GFAP^+^ astrocytes in pKr-2-injected CA1 layer of hippocampus in vivo (Figure 6J).

To assess the extent of ROS/RNS damages on proteins (protein nitration), we measured the levels of nitrotyrosine in the absence or presence of IL-4Nab. Immunohistochemical analysis revealed the significant increase in nitrotyrosine levels in the CA1 layer of hippocampus at 3 days after pKr-2 injection (Figure 6H,M) compared to PBS-injected control (Figure 6G,M). Treatment with IL-4Nab attenuated levels of pKr-2-induced nitrotyrosine in the CA1 layer of hippocampus in vivo (Figure 6I,M).

## 4. Discussion

Oxidative/nitrosative stress contributes to increased levels of ROS/RNS formation that emerge as a crucial factor for neurodegeneration in vivo and in vitro [1,2,4]. iNOS is one of major source for excessive production of ROS/RNS, resulting in oxidative damage to proteins, lipids and DNA in AD [2,3]. Accumulating experimental evidence, including ours, showed that intracortical or intrahippocampal injection of thrombin, pKr-2, LPS and beta-amyloid_1-42_ increased the iNOS-derived ROS/RNS production in vivo [9,21,28,29,30]. The results of the present study demonstrate that neutralization of IL-4 significantly decreases pKr-2-induced ROS production and oxidative damages on DNA and protein and prevents pKr-2-induced degeneration of hippocampal neurons in vivo. This is similar to our previous studies showing that neutralization of IL-4 attenuated ROS production and prevented pKr-2-, thrombin- and LPS-induced degeneration of cortical neurons in vivo [9,18,22]. It is therefore likely that IL-4 might function as proinflammatory mediator in pKr-2-lesioned hippocampus in vivo through production of iNOS-derived ROS/RNS.

Accompanying microglial iNOS-derived ROS/RNS, several lines of evidence demonstrate that MPO-derived ROS/RNS can trigger neuronal death-related signaling cascades in vivo [4,31,32]. MPO is the key enzyme involved in the generation of cytotoxic ROS/RNS [32,33,34], resulting in oxidative damages to proteins, lipids and DNA in neurodegenerative disorders. Several reports, including ours, have demonstrated that various antioxidants, such as glutathione, ethyl pyruvate and capsaicin attenuated MPO-derived ROS/RNS levels and oxidative damages in vivo and in vitro [31,35,36]. Our results showed that accompanying reduction of ROS/RNS levels and oxidative damages, neutralization of IL-4 significantly attenuated MPO expression and neurodegeneration in pKr-2-injected hippocampus in vivo. This indicates that like the iNOS, production of MPO-derived ROS/RNS might be regulated by IL-4. The present results have demonstrated that IL-4Nab partially, not fully attenuated oxidative stress markers such as iNOS/MPO. This may be a concentration effect as only one dose of IL-4Nab was used here, indicating that IL-4 is not the only factor to regulate iNOS/MPO under our current experimental conditions.

MPO is expressed in astrocytes, microglia/macrophages and neutrophil under neuropathological conditions [4,25,31,32,35]. In the present study, we demonstrate that MPO is colocalized with OX-42^+^ microglia/macrophages, but not in GFAP^+^ astrocytes in the CA1 layer of pKr-2-injected rat hippocampus in vivo. This is inconsistent with previous reports showing that MPO was expressed in GFAP^+^ astrocytes, but not in reactive microglia in the substantia nigra of 1-methyl-4-phenyl-1,2,3,6-tetrahydropyridine (MPTP) mice [26,31,35]. This discrepancy may reflect differences in toxins used (pKr-2 versus MPTP), regions studies (hippocampus versus substantia nigra) and/or animals (rat versus mouse) in each experiment. This interpretation is supported by previous findings that MPO is expressed in neutrophil, but not in astrocytes in mice exposed to spinal cord injury [37]. However, it cannot exclude the possibility that in pKr-2-injected hippocampus, MPO is expressed in neutrophil because of absence of distinguishable marker between microglia/macrophages and neutrophil.

Moreover, microglia are able to acquire either proinflammatory phase (M1) or anti-inflammatory phase (M2) during neuroinflammation [38,39]. In the present study, immunohistochemical analysis showed that IL-4Nab inhibited both microglia activation and iNOS/MPO expression within microglia. These results suggest that IL-4 might play a dual action such as microglial activation and/or polarization in pKr-2-lesioned hippocampus in vivo.

## 5. Conclusions

In summary, the major finding of this manuscript is the observation that endogenous IL-4 expressed within reactive microglia/macrophages aggravates pKr-2-induced neurodegeneration of the rat hippocampal CA1 area in vivo. This occurs through IL-4-induced oxidative stress since IL-4 neutralization reduces ROS/RNS production, number of iNOS^+^ and MPO^+^ reactive microglia/macrophages and oxidative damages, resulting in neuroprotection of pKr-2-treated hippocampus CA1 area in vivo. Thus, our results suggest that IL-4 contributes to the neurodegenerative processes in which neuroinflammation and oxidative stress are implicated.

## Figures and Tables

**Table 1:** Primary antibodies used for immunohistochemistry (IHC) and immunofluorescence (IF).

Primary Antibody	Dilution	Company	Catalog No.
**OX-42**	1:400	Bio-rad	MCA275G
**OX-6**	1:400	BD Biosciences	554926
**FITC-TL**	1:1000	Vector Laboratories	FL-1171
**NeuN**	1:1000	Merck	MAB377
**GFAP**	1:500	Sigma-Aldrich	G3893
**IL-4**	1:400	Abbiotec	251223
**MPO**	1:500	DakoCytomation	A0398
**iNOS**	1:200	BD Biosciences	610333
**8-OHdG**	1:300	Jaica	MOG-100P
**Nitrotyrosine**	1:50	Abcam	ab7048

**Table 2:** Secondary antibodies used for IHC and IF.

Secondary Antibody	Dilution	Company	Catalog No.
**Biotin-conjugated anti-mouse IgG**	1:400	KPL	16-18-15
**FITC-conjugated anti-mouse IgG**	1:500	Sigma-Aldrich	AP124F
**Fluorescein-conjugated anti-mouse IgG**	1:300	Vector Laboratories	FI-2000
**Cy3-conjugated anti-rabbit IgG**	1:1000	Sigma-Aldrich	AP132C
